# Supplementary material for: PLA2G12A as a Novel Biomarker for Colorectal Cancer with Prognostic Relevance
Source: Int J Mol Sci. 2023 Jun 30;24(13):10889. doi: 10.3390/ijms241310889 (PMC10341627; doi:10.3390/ijms241310889)
Supplement: Supplementary file 1 [file ijms-24-10889-s001.zip › ijms-2397922-supplementary.pdf]

Supplementary Table S1. *Drosophila melanogaster* genetic screen data

Gene	CTC	SEM	*p*	TB	SEM	*p*	Gene	CTC	SEM	*p*	TB	SEM	*p*
**Control**	100			100	4		**Control**	100			100	4	
**Aats-trp**	ut			14	0	**0,000**	**Gap1**	522	55	**0,003**	497	66	**0,001**
**Alph**	ut			170	7	**0,002**	**Gs1**	109	33	0,667	219	10	**0,005**
**AmyD**	ut			130	4	0,052	**GXIVsPLA2**	697	16	**0,014**	305	25	**0,007**
**ap (apterous)**	ut			299	36	**0,005**	**Hh**	178	33	0,283	455	40	**0,005**
**aru**	130	7	0,352	371	23	**0,005**	**Hsc70-4**	ut			13	2	**0,002**
**bai**	2541	144	**0,000**	476	93	0,000	**Iap2**	168	16	0,065	118	7	0,838
**beat1b**	ut			121	10	0,272	**Idgf2**	ut			324	8	**0,000**
**Bnl**	411	74	0,100	140	5	**0,022**	**ImpL2**	603	63	**0,004**	383	37	**0,001**
**br**	192	10	0,196	162	10	**0,046**	**Ipk2**	298	13	**0,011**	270	15	**0,001**
**brwd3**	188	4	0,063	257	10	**0,005**	**Irc**	ut			62	3	**0,020**
**bw**	221	31	**0,045**	341	35	**0,005**	**irk2**	ut			159	3	**0,026**
**Ca-beta**	ut			82	3	0,921	**Itgbn**	ut			216	16	0,067
**Cactin**	115	14	0,352	55	3	**0,008**	**jhamt**	193	9	0,067	108	8	0,542
**CAH-2**	ut			108	4	0,202	**klu**	ut			127	5	0,065
**Cdc42**	121	9	0,275	137	6	**0,048**	**kon**	241	40	0,542	159	18	0,077
**ced6**	416	153	0,497	206	18	0,905	**lab**	122	12	0,539	169	24	0,539
**CG1021**	365	27	**0,047**	146	4	**0,019**	**LKB1**	1944	292	**0,039**	201	60	0,097
**CG10916**	ut			98	5	0,841	**loco**	ut			199	13	**0,025**
**CG11125**	ut			164	5	**0,009**	**luna**	ut			47	2	**0,001**
**CG11147**	138	3	0,328	50	2	**0,001**	**Magu**	137	11	0,360	139	5	**0,041**
**CG11188**	ut			100	4	0,499	**Mal-A1**	290	12	**0,011**	96	7	0,278
**CG11778**	ut			140	3	**0,000**	**mct1**	ut			36	2	**0,002**
**CG13623**	167	18	0,316	155	6	**0,012**	**Mlh1**	115	14	0,792	121	18	0,752
**CG16749**	ut			253	27	0,430	**mmp1**	ut			156	30	0,268
**CG17187**	ut			32	2	**0,000**	**Msh6**	812	88	0,300	414	14	**0,002**
**CG17188**	381	17	0,112	171	18	**0,021**	**mthl2**	ut			200	5	**0,003**
**CG17633**	ut			244	7	**0,000**	**mtSSB**	169	10	0,306	144	8	0,106
**CG18278**	ut			160	6	**0,008**	**mtTFB2**	156	14	0,569	100	9	0,741
**CG31055**	112	14	0,569	153	7	**0,021**	**nonA**	379	11	**0,029**	36	3	**0,004**
**CG31704**	ut			91	8	0,714	**pros54**	124	13	0,881	160	13	0,112
**CG32369**	163	5	0,219	68	4	0,375	**Pvr**	133	11	0,428	113	6	0,113
**CG32549**	ut			50	6	**0,015**	**RanGap**	217	27	0,785	11	1	**0,002**
**CG33181**	ut			37	1	**0,009**	**Rock**	ut			226	12	**0,009**
**CG40485**	ut			204	5	**0,000**	**sens**	106	20	0,245	128	13	0,946
**CG42666**	ut			25	3	**0,001**	**sesB**	151	16	0,547	106	7	0,633
**CG44008**	ut			97	2	0,152	**smo**	233	94	0,426	543	87	**0,000**
**CG45263**	ut			172	19	0,200	**SPARC**	ut			114	3	0,661
**CG4830**	ut			248	13	**0,005**	**Src42**	201	23	**0,009**	237	51	**0,002**
**CG5390**	211	19	0,115	154	16	0,086	**stc**	456	11	**0,039**	227	15	**0,002**
**CG5550**	ut			156	18	0,356	**stl**	160	11	0,569	155	9	**0,015**
**CG6287**	ut			78	5	0,062	**tau**	548	50	**0,007**	94	4	0,390
**CG9098**	1108	31	**0,018**	306	5	**0,006**	**Theta-Try**	ut			26	1	**0,000**
**cont**	890	56	**0,009**	189	13	**0,029**	**tobi**	ut			179	4	0,539
**CrebA**	475	45	**0,032**	173	28	**0,036**	**Tsp**	384	28	**0,022**	218	40	0,058
**da**	ut			20	1	**0,000**	**Ugt36Ba**	ut			8	0	**0,006**
**Dl**	793	4	**0,001**	432	57	**0,001**	**veil**	178	2	0,107	116	5	0,472
**Dp110**	332	10	**0,011**	219	12	**0,009**	**Vha100-4**	527	54	0,712	127	2	0,140
**eta try**	ut			165	3	**0,006**	**Zfh1**	ut			15	1	**0,004**
**Fur1**	ut			200	10	**0,001**							

Data points obtained from the luciferase analysis represent averages of at least 3 replicates of batches of 10 individuals. Light intensity measurement is an indicator of the circulating tumor cells (CTCs) number and the total tumor burden (TB) and are represented as percentage over the control Apc-Ras. In the case of CTCs, value of the control Apc-Ras is the minimum value detected by the assay, so it was considered as a threshold. Values under the threshold are labeled ut. Significant values with $p < 0.05$ obtained by the Mann-Whitney U test are shown in bold.

Supplementary Table S2. Genes selected with the Drosophila CRC model

Gene	Original *p* value	Benjamini-Hochberg adjusted *p* value
**bai**	0,000	0,000
**Dl**	0,001	0,033
**Gap1**	0,003	0,050
**ImpL2**	0,004	0,057
**tau**	0,007	0,070
**cont**	0,009	0,082
**Src42**	0,009	0,075
**Ipk2**	0,011	0,073
**Mal-A1**	0,011	0,065
**Dp110**	0,011	0,061
**GXIVsPLA2**	0,014	0,074
**CG9098**	0,018	0,086
**Tsp**	0,022	0,092

Selected genes with a significant *p* value obtained by the Mann-Whitney U test and corrected by the Benjamini-Hochberg procedure.

Supplementary Table S3. Hazard ratios (HR) for Recurrence Free Survival (RFS) and Overall Survival (OS)

**Human Gene**	**RFS**			**OS**		
	**HR**	**95% CI**	***p***	**HR**	**95% CI**	***p***
CNTN4	1,210	0,868 - 1,688	0,260	1,013	0,702 - 1,463	0,945
DLL1	1,098	0,790 - 1,526	0,577	1,102	0,765 - 1,589	0,602
FRK	0,784	0,564 - 1,089	0,147	0,857	0,594 - 1,236	0,409
IPMK	0,794	0,570 - 1,106	0,173	0,761	0,524 - 1,107	0,153
PIK3CD	1,151	0,828 - 1,602	0,403	1,149	0,795 - 1,661	0,460
PLA2G12A	0,580	0,414 - 0,813	**0,002**	0,699	0,483 - 1,011	0,057
RASA3	1,108	0,797 - 1,540	0,541	1,038	0,720 - 1,497	0,840
SH2D3C	1,255	0,903 - 1,746	0,177	1,148	0,796 - 1,656	0,461
SLC3A1	0,767	0,551 - 1,067	0,116	0,963	0,662 - 1,400	0,842
TMED10	0,929	0,669 - 1,292	0,662	0,900	0,625 - 1,298	0,574

RNA expression high *vs* low. HR < 1 indicating low expression poor prognosis. HR > 1 indicating high expression poor prognosis. Significant values obtained by the Cox proportional hazards model with $p < 0.05$ are shown in bold.

Supplementary Table S4. Top hallmark gene signatures associated with *PLA2G12A* expression in the TCGA cohort

GS DETAILS	SIZE	ES	NES	NOM *p*-val
HALLMARK_EPITHELIAL_MESENCHYMAL_TRANSITION	194	-0,64	-1,78	**0,012**
HALLMARK_INTERFERON_GAMMA_RESPONSE	196	-0,55	-1,61	0,092
HALLMARK_INTERFERON_ALPHA_RESPONSE	92	-0,55	-1,45	0,173
HALLMARK_APICAL_JUNCTION	189	-0,51	-1,87	**0,002**
HALLMARK_ANGIOGENESIS	36	-0,51	-1,47	0,093
HALLMARK_MYOGENESIS	185	-0,5	-1,72	**0,014**
HALLMARK_HEDGEHOG_SIGNALING	34	-0,48	-1,41	0,091
HALLMARK_NOTCH_SIGNALING	32	-0,47	-1,63	**0,01**
HALLMARK_APICAL_SURFACE	42	-0,46	-1,5	0,056
HALLMARK_ALLOGRAFT_REJECTION	188	-0,45	-1,39	0,196
HALLMARK_COAGULATION	124	-0,44	-1,55	0,054
HALLMARK_COMPLEMENT	192	-0,41	-1,48	0,094
HALLMARK_INFLAMMATORY_RESPONSE	195	-0,41	-1,34	0,195
HALLMARK_WNT_BETA_CATENIN_SIGNALING	42	-0,4	-1,32	0,172
HALLMARK_IL6_JAK_STAT3_SIGNALING	85	-0,4	-1,27	0,231
HALLMARK_IL2_STAT5_SIGNALING	192	-0,36	-1,42	0,1
HALLMARK_KRAS_SIGNALING_DN	155	-0,35	-1,35	0,067
HALLMARK_REACTIVE_OXYGEN_SPECIES_PATHWAY	45	-0,35	-1,19	0,253
HALLMARK_KRAS_SIGNALING_UP	187	-0,34	-1,31	0,186
HALLMARK_HYPOXIA	187	-0,33	-1,26	0,215

Significant values with $p < 0.05$ are shown in bold.

Supplementary Table S5. Baseline characteristics of the internal CRC HUAV cohort

**Characteristic**		
**Sex**	Male (%)	114 (63.3)
	Female (%)	66 (36.7)
**Age**	Median years (range)	75.4 (37.2 - 92.4)
**Anatomic location**	Cecum (%)	13 (7.2)
	Ascending colon (%)	49 (27.2)
	Hepatic flexure (%)	4 (2.2)
	Transverse colon (%)	4 (2.2)
	Splenic flexure (%)	5 (2.8)
	Descending colon (%)	11 (6.1)
	Sigmoid colon (%)	61 (33.9)
	Rectosigmoid junction (%)	6 (3.3)
	Rectum (%)	27 (15)
**Stage**	I (%)	8 (4.4)
	II (%)	58 (32.2)
	III (%)	114 (63.3)
**Histology**	Adenocarcinoma (%)	151 (83.9)
	Mucinous adenocarcinoma (%)	26 (14.4)
	Signet ring cell carcinoma (%)	2 (1.1)
	Medullary carcinoma (%)	1 (0.6)
**Differentiation degree**	Low-grade (%)	150 (83.3)
	High-grade (%)	30 (16.7)
**Venous invasion** [missing 1]	Yes (%)	63 (35.2)
	No (%)	116 (64.8)
**Lymphatic invasion** [missing 3]	Yes (%)	69 (39)
	No (%)	108 (61)
**Perineural invasion** [missing 4]	Yes (%)	55 (31.3)
	No (%)	121 (68.8)
**Microsatellite status** [missing 96]	MSS (%)	79 (94)
	MSI (%)	5 (6)
**RAS** [missing 145]	RAS mut (%)	20 (57.1)
	RAS wt (%)	15 (42.9)
**Adjuvant chemotherapy**	Yes (%)	99 (55)
	No (%)	81 (45)

Supplementary Figure S1

A

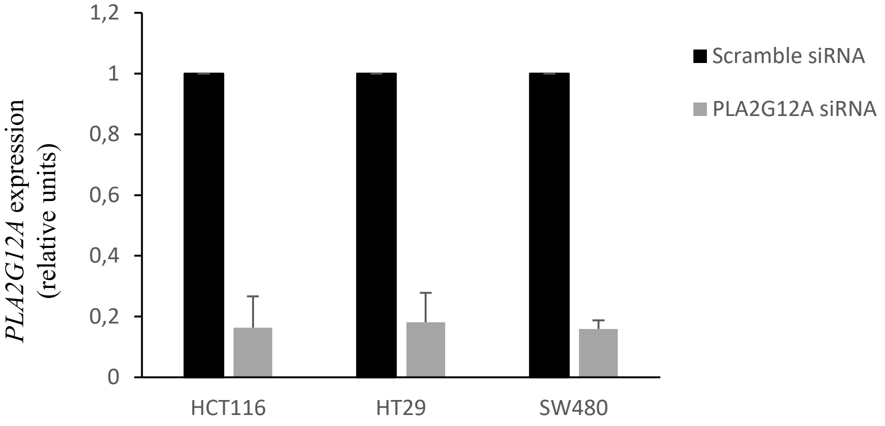

B

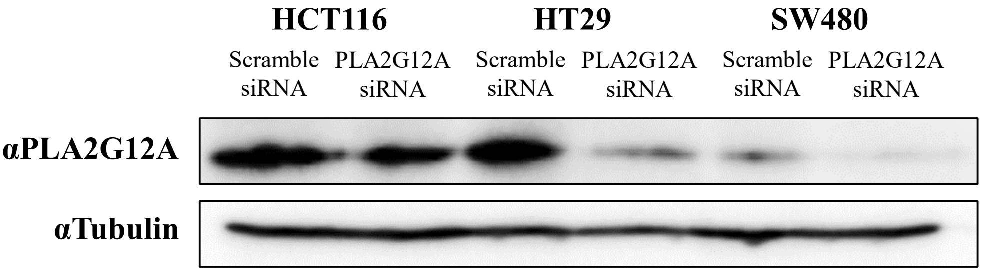

Supplementary Figure S1. *PLA2G12A* expression decreased when *PLA2G12A* was downregulated via siRNA. **A.** Relative mRNA expression levels were calculated at 48h post-transfection using the $2^{-\Delta\Delta Ct}$ method with β-actin as a housekeeping gene control in three different CC cell lines, HCT116, HT29 and SW480. Relative quantitative values are shown as mean ± SEM of three biological replicates, each of them containing three technical replicates. **B**. Representative western blot of PLA2G12A protein levels 48h after siRNA transfection. The membranes were cut so that only the portion of gel containing the desired bands would be visualized. The samples were from the same experiment and the blots were processed in parallel.
